# Phylogenomic Relationships between Amylolytic Enzymes from 85 Strains of Fungi

**DOI:** 10.1371/journal.pone.0049679

**Published:** 2012-11-15

**Authors:** Wanping Chen, Ting Xie, Yanchun Shao, Fusheng Chen

**Affiliations:** 1 National Key Laboratory of Agro-Microbiology, Huazhong Agricultural University, Wuhan, Hubei Province, China; 2 Key Laboratory of Environment Correlative Dietology (Ministry of Education), Huazhong Agricultural University, Wuhan, Hubei Province, China; 3 College of Food Science and Technology, Huazhong Agricultural University, Wuhan, Hubei Province, China; Oregon State University, United States of America

## Abstract

Fungal amylolytic enzymes, including α-amylase, gluocoamylase and α-glucosidase, have been extensively exploited in diverse industrial applications such as high fructose syrup production, paper making, food processing and ethanol production. In this paper, amylolytic genes of 85 strains of fungi from the phyla Ascomycota, Basidiomycota, Chytridiomycota and Zygomycota were annotated on the genomic scale according to the classification of glycoside hydrolase (GH) from the Carbohydrate-Active enZymes (CAZy) Database. Comparisons of gene abundance in the fungi suggested that the repertoire of amylolytic genes adapted to their respective lifestyles. Amylolytic enzymes in family GH13 were divided into four distinct clades identified as heterologous α- amylases, eukaryotic α-amylases, bacterial and fungal α-amylases and GH13 α-glucosidases. Family GH15 had two branches, one for gluocoamylases, and the other with currently unknown function. GH31 α-glucosidases showed diverse branches consisting of neutral α-glucosidases, lysosomal acid α-glucosidases and a new clade phylogenetically related to the bacterial counterparts. Distribution of starch-binding domains in above fungal amylolytic enzymes was related to the enzyme source and phylogeny. Finally, likely scenarios for the evolution of amylolytic enzymes in fungi based on phylogenetic analyses were proposed. Our results provide new insights into evolutionary relationships among subgroups of fungal amylolytic enzymes and fungal evolutionary adaptation to ecological conditions.

## Introduction

Starch is the major carbohydrate storage product of green plants as a result of photosynthesis and makes up an important part of carbon and energy sources widely consumed among animals, plants and microorganisms [1]–[3]. Besides its direct use as a food source, starch is also utilized as a raw material in many industrial applications such as the production of ethanol, glues, high fructose syrups and paper [1], [3]. Starch consists of two types of glucose polymers: (i) amylose, a linear polymer of glucose residues linked by α-1,4-glycosidic bonds and (ii) amylopectin, an α-1,4-linked D-glucan with varying proportions of α-1,6-linked branches [1], [3], [4]. The potential of starch as a renewable biological resource has stimulated research into amylolytic enzymes.

As heterotrophic microorganisms, fungi utilize polysaccharide substrates through a complement of hydrolytic enzymes secreted into the environmental niches to digest large organic molecules into smaller molecules that may then be absorbed as nutrients. Some fungi, for example members of the genus *Aspergillus* with high yields of powerful amylolytic enzymes have been extensively exploited for industrial applications [1], [2], [5]–[7]. Fungi generally produce three types of amylolytic enzymes: α-amylase (EC 3.2.1.1), glucoamylase (EC 3.2.1.3) and α-glucosidase (EC 3.2.1.20) [8]–[11]. Based on the classification of glycoside hydrolase (GH) from the Carbohydrate-Active enZymes (CAZy) Database (http://www.cazy.org) [12], the vast majority of these amylolytic enzymes are divided into the GH13, GH15 and GH31 families.

α-amylases act on α-1,4-glycosidic bonds with the endo-hydrolysis of the long polysaccharide chains into shorter maltooligosaccharides and α-limit dextrins [10], [13], [14]. Commercial applications of α-amylases from fungi such as representative strains of *Aspergillus niger* and *A. oryzae* are numerous and the largest volume is considered to be used for thinning of starch in the liquefaction process in the sugar, alcohol and brewing industries [5], [15]. Currently, α-amylases are unambiguously found in families GH13, GH57 and GH119 [16]. However, α-amylases in families GH57 and GH119 are solely from prokaryotes at present [16], [17]. Family GH13 is the major α-amylase family consisting of more than 30 different enzyme specificities and together with GH70 and GH77 forms the clan GH-H [1], [12]. Members of clan GH-H share a (β/α)_8_ barrel domain and can be recognized by 4–7 conserved amino acid regions containing three catalytic residues, which are believed to represent a common evolutionary origin [16], [18]–[20]. The phylogeny of GH13 α-amylases is generally in agreement with their origin. For example, all fungal α-amylases are more related to each other than to the α-amylases originating from plants or animals. α-amylases from bacteria, however, are scattered over several clusters, which group with animal, plant or fungal α-amylases can be explained as the results of horizontal gene transfer from Eukarya to Bacteria [13], [14], [21], [22]. At present, fungal α-amylases are classified into two subfamilies GH13_1 and GH13_5 [1]. Members in subfamily GH13_1 are extracellular and fungal specific, while those in subfamily GH13_5 are intracellular and have high sequence similarities to the bacterial α-amylases [13].

Glucoamylases, also known as γ-amylases, catalyse hydrolysis of α-1,4 and α-1,6 glucosidic linkages to release β-D-glucose from the non-reducing ends of starch and related poly- and oligosaccharides [10], [23], [24]. Industrially glucoamylases are produced from filamentous fungi, *Aspergillus* spp. and *Rhizopus* spp., whose major commercial application (“starch saccharification”) is to break down starch to yield glucose for use in food and fermentation industries [5], [15], [23], [25], [26]. For instance, glucoamylase is widely applied in fermentation industries of traditional foods such as sake, shoyu and miso in Asian countries [27], [28]. Glucoamylases are found solely in family GH15 [29]. Catalytic domains of most glucoamylases share the same architecture, being comprised of thirteen helices of which twelve form an (α/α)_6_ barrel [23], [25]. Glucoamylases occur in some prokaryotic and many eukaryotic microorganisms, and may have originated as a polysaccharide exo-hydrolase early in the evolution of glycogen metabolism [26].

α-glucosidases hydrolyze α-1,4 and/or α-1,6-linkages of saccharides to liberate α-D-glucose from the non-reducing end [5], [10], [30], [31]. α-glucosidases for commercial use are produced from *Aspergillus* spp. and *Mucor* spp. [15]. At present, α-glucosidases are found in four families: GH4, GH13, GH31 and GH97 [32]. α-glucosidases from family GH31 are the most widespread and can be found in all three domains of life [30]. The enzymes from GH13 originate from bacteria, and in eukaryotes are limited to fungi and insect, while those from families GH4 and GH97 are solely of bacterial origin [32]. α-glucosidases from families GH13 and GH31 share a (β/α)_8_ barrel fold of their catalytic domain, and a remote but significant homology between the two GH families suggests a common ancestor [33], [34].

Amylolytic enzymes of microorganisms, in particular filamentous fungi, from the families GH13 and GH15 often possess starch-binding domains facilitating attachment and degradation of raw starch [35]–[37]. These domains are very frequently positioned at the C-terminal end of enzymes, and some exceptions such as the *R. oryzae* glucoamalyse present their starch-binding domains at the N-terminus [35], [38], [39]. Currently, starch-binding domains are categorized into ten carbohydrate-binding module (CBM) families 20, 21, 25, 26, 34, 41, 45, 48, 53 and 58 based on their amino acid sequence similarities in the CAZy database [40], [41]. Among them, CBM20 family is the most generalized and studied family [37], [38]. Phylogenetic analysis revealed that starch-binding domain might be an independent module and showed a separate evolution, which reflected the evolution of their origin rather than the individual amylases [36], [42].

Fungal amylolytic enzymes as the major industrial source play an important role in starch processing. There have been extensive studies focused on the identification and regulation of fungal amylolytic genes [2]. However, researches with respect to distribution, abundance and phylogeny of amylolytic genes have been less common. The availability of whole genome sequences for a number of fungi opens new research avenues to reach a global understanding of problems concerning the relationships between genomic characteristics and fungal lifestyles. In this study, the genome sequences of 85 strains of fungi from the four traditionally recognized phyla Ascomycota, Basidiomycota, Chytridiomycota and Zygomycota were surveyed to identify related GH13, GH15 and GH31 family members with hidden Markov models. Additionally, we have analyzed the phylogeny of these proteins, the presence of specific protein features, the distribution of starch-binding domains and synteny among these fungal species, which allowed division of the members of each GH family into several groups. Based on the phylogenetic analyses, we propose possible evolutionary events and hypothetical scenarios for the evolution of amylolytic enzymes in fungi.

## Results/Discussion

### Genomic Distribution of Amylolytic Genes in the Tested Fungi Adapts to their Lifestyles in Starch Degradation

Putative amylolytic genes from 85 strains of fungi were identified by HMMER searches and numbers of the annotated amylolytic genes were compared among these fungi (**Table 1**). The annotation results showed that phylogenetically close species shared similar numbers for each enzyme class. Genes of glucoamylases and GH31 α-glucosidases were found in all tested fungi from the phyla Ascomycota, Basidiomycota, Chytridiomycota and Zygomycota, which inferred that glucoamylases and α-glucosidases were the vital enzymes for fungi, probably due to glucose as a major source of energy in fungi. Loss of such enzymes may be not conducive for fungi to obtain glucose by hydrolyzing the main storage polysaccharide–starch. However, the amylolytic genes from the family GH13, including α-amylases and α-glucosidases (GH13), were not positively identified in some species, and thus seem to be non-essential in fungi compared to glucoamylases and α-glucosidases (GH31).

**Table 1:** Distribution of putative genes involved in starch degradation in 85 fungal genomes.

Phylum	Taxonomic group	Species	Strains	Abbreviation	α-amylases(GH13)	GH15	α-glucosidases(GH13)	α-glucosidases(GH31)
Ascomycota	Dothideomycetes	*Leptosphaeria maculans*	JN3	Lm	2	3	3	5
		*Phaeosphaeria nodorum*	SN15	Pn	2	3	3	7
		*Zymoseptoria tritici*	IPO323	Zt	5	1	4	7
	Eurotiales	*Aspergillus clavatus*	NRRL 1	Acl	7	6	4	3
		*A. flavus*	NRRL 3357	Afl	5	3	5	6
		*A. fumigatus*	Af293	Afu	6	5	4	4
		*A. kawachii*	IFO 4308	Ak	8	2	1	5
		*A. niger*	CBS 513.88	An	6	2	2	4
		*A. oryzae*	RIB40	Aor	6	2	5	5
		*A. terreus*	NIH2624	At	7	2	2	6
		*Emericella nidulans*	FGSC A4	En	7	2	2	5
		*Monascus ruber*	M7	Mr	3	3	4	2
		*Neosartorya fischeri*	NRRL 181	Nf	7	5	4	6
		*Penicillium chrysogenum*	Wisconsin54-1255	Pc	7	3	4	8
		*P. marneffei*	ATCC 18224	Pm	5	4	1	5
		*Talaromyces stipitatus*	ATCC 10500	Ts	5	4	3	5
	Onygenales	*Ajellomyces capsulatus*	G186AR	Aca	3	1	0	3
		*Arthroderma otae*	CBS 113480	Aot	1	1	0	2
		*Coccidioides immitis*	RS	Ci	2	1	0	2
		*C. posadasii*	str. Silveira	Cp	2	1	0	2
		*Paracoccidioides brasiliensis*	Pb01	Pb	3	1	0	2
		*Trichophyton equinum*	CBS 127.97	Te	1	1	0	2
		*T. rubrum*	CBS 118892	Tr	1	1	0	2
		*T. tonsurans*	CBS 112818	Tto	1	1	0	2
		*T. verrucosum*	HKI 0517	Tv	1	1	0	2
	Orbiliomycetes	*Arthrobotrys oligospora*	ATCC 24927	Aol	2	4	2	2
	Pezizomycetes	*Tuber melanosporum*	Mel28	Tm	4	1	1	3
	Saccharomycotina	*Candida albicans*	WO-1	Ca	0	3	2	1
		*C.dubliniensis*	CD36	Cd	0	3	2	1
		*C. glabrata*	CBS 138	Cga	0	1	0	1
		*C. tropicalis*	MYA-3404	Ct	0	2	5	1
		*Clavispora lusitaniae*	ATCC 42720	Cl	0	1	2	2
		*Debaryomyces hansenii*	CBS767	Dh	0	2	2	1
		*Eremothecium cymbalariae*	DBVPG#7215	Ec	0	1	0	1
		*E. gossypii*	ATCC 10895	Eg	0	1	0	1
		*Kluyveromyces lactis*	NRRL Y-1140	Kl	0	1	2	1
		*Komagataella pastoris*	CBS 7435	Kp	0	1	0	1
		*Lachancea thermotolerans*	CBS 6340	Lt	0	1	4	1
		*Lodderomyces elongisporus*	NRRL YB-4239	Le	0	1	1	1
		*Meyerozyma guilliermondii*	ATCC 6260	Mg	0	1	3	1
		*Naumovozyma castellii*	CBS 4309	Nca	0	1	0	1
		*N. dairenensis*	CBS 421	Nd	0	1	0	1
		*Ogataea parapolymorpha*	DL-1	Op	0	1	1	1
		*Saccharomyces cerevisiae*	YJM789	Sce	0	1	3	1
		*Scheffersomyces stipitis*	CBS 6054	Sst	0	2	5	2
		*Tetrapisispora phaffii*	CBS 4417	Tp	0	1	0	1
		*Torulaspora delbrueckii*	CBS 1146	Td	0	1	3	1
		*Yarrowia lipolytica*	CLIB122	Yl	0	1	0	1
		*Zygosaccharomyces rouxii*	CBS 732	Zr	0	1	0	2
	Sordariomyceta	*Botryotinia fuckeliana*	B05.10	Bf	6	4	0	3
		*Chaetomium globosum*	CBS 148.51	Cgo	4	2	2	3
		*Cordyceps militaris*	CM01	Cm	1	2	1	3
		*Gibberella zeae*	PH-1	Gz	1	3	5	3
		*Glarea lozoyensis*	74030	Gl	2	2	2	2
		*Glomerella graminicola*	M1.001	Gg	5	3	3	4
		*Grosmannia clavigera*	kw1407	Gc	0	2	1	1
		*Hypocrea jecorina*	QM6a	Hj	1	2	2	3
		*Magnaporthe oryzae*	70–15	Mo	4	2	1	3
		*Metarhizium acridum*	CQMa 102	Mac	1	2	3	4
		*M. anisopliae*	ARSEF 23	Man	1	2	3	4
		*Myceliophthora thermophila*	ATCC 42464	Mt	3	2	2	3
		*Nectria haematococca*	mpVI 77-13-4	Nh	1	2	5	4
		*Neurospora crassa*	OR74A	Ncr	4	2	2	4
		*N. tetrasperma*	FGSC 2508	Nt	4	2	2	4
		*Sclerotinia sclerotiorum*	1980 UF-70	Ssc	6	4	1	3
		*Sordaria macrospora*	k-hell	Sm	5	3	2	4
		*Thielavia terrestris*	NRRL 8126	Tte	1	3	2	4
		*Verticillium albo-atrum*	VaMs.102	Va	2	4	3	3
		*V. dahlia*	VdLs.17	Vd	2	4	3	4
	Taphrinomycotina	*Schizosaccharomyces japonicus*	yFS275	Sj	6	2	0	3
		*S. pombe*	972h-	Sp	7	2	1	4
Basidiomycota	Agaricomycotina	*Coprinopsis cinerea*	okayama7#130	Cc	4	4	2	3
		*Cryptococcus gattii*	WM276	Cgt	5	2	2	3
		*C. neoformans*	var. neoformans B-3501A	Cn	5	2	2	3
		*Laccaria bicolor*	S238N-H82	Lb	4	2	1	3
		*Moniliophthora perniciosa*	FA553	Mp	2	3	0	4
		*Postia placenta*	Mad-698-R	Pp	2	2	0	6
		*Schizophyllum commune*	H4-8	Sco	8	3	1	3
		*Serpula lacrymans*	var. lacrymans S7.3	Sl	3	2	1	4
	Pucciniomycotina	*Melampsora larici-populina*	98AG31	Ml	3	4	0	3
		*Puccinia graminis f. sp. tritici*	CRL 75-36-700-3	Pg	1	3	0	2
	Ustilaginomycotina	*Sporisorium reilianum*	SRZ2	Sr	1	1	2	3
		*Ustilago maydis*	1	Um	1	1	1	3
Chytridiomycota	Chytridiomycetes	*Batrachochytrium dendrobatidis*	JAM81	Bd	0	1	0	2
Zygomycota	Mucoromycotina	*Rhizopus oryzae*	RA 99-880	Ro	1	6	0	2

Taxonomy information of above fungi is extracted from Taxonomy Browser in NCBI (http://www.ncbi.nlm.nih.gov/Taxonomy/CommonTree/wwwcmt.cgi). Overall protein sequences of *Rhizopus oryzae* and *Ustilago maydis* were downloaded from the Broad Institute (http://www.broadinstitute.org/scientific-community/data), *Monascus ruber* was from our lab and others were from genome resource of NCBI (http://www.ncbi.nlm.nih.gov/genome/browse/). Putative amylolytic enzymes from the GH13, GH15 and GH31 families in each species were inferred by searching its overall proteins with the corresponding profile hidden Markov models from Pfam (http://pfam.sanger.ac.uk/) and their annotations followed by BlastP comparisons against the database of non-redundant protein sequences (http://blast.ncbi.nlm.nih.gov/Blast.cgi). Accession numbers of putative proteins were shown in the corresponding phylogenetic trees later. Some proteins from the family GH13 with equivocal assignment between α-amylases and α-glucosidases were assigned to the group of α-glucosidase (GH13).

The distribution of amylolytic genes from the tested fungi also suggested a strong relationship between the repertoire of amylolytic enzymes in fungal genomes and their saprophytic lifestyle. Members of the genus *Aspergillus* such as *A. oryzae* and *A. niger* are known as strong producers of amylolytic enzymes, which have been widely exploited for commercial use [2]. *Monascus* spp. and *Penicillium* spp. are also notable for their amylolytic enzyme production and widely used in food processing [28]. Accordingly, fungal genomes from Eurotiales were identified as the taxa with the high abundance of amylolytic genes. However, fungal genomes from Onygenales, which are close relatives of Eurotiales in taxonomy, owned low numbers of amylolytic genes and had no positively identified α-glucosidases (GH13). Ascomycota fungi from group Dothideomycetes, Orbiliomycetes, Pezizomycetes, Sordariomyceta and Taphrinomycotina, most of which are plant pathogens, are also rich in amylolytic enzymes. It is worth noting that members from Saccharomycotina possessed low abundance of amylolytic genes and no α-amylase was positively indentified. As reflected in their biological characteristics, the yeasts from Saccharomycotina lack the ability to utilize raw starch as a carbon source and the notable example is *Saccharomyces cerevisiae*, the main organism used for alcoholic fermentation but limited in starch hydrolysis [43]–[45]. This implies that the α-amylase genes were likely to be lost in the clade of Saccharomycotina during the evolution.

For the phylum of Basidiomycota, fungi from Agaricomycotina had more abundance than those from Pucciniomycotina and Ustilaginomycotina in amylolytic gene distribution. *Rhizopus oryzae*, as the representative filamentous fungus from the phylum Zygomycota, is used in the production of various fermented foods and alcoholic beverages in several Asian countries (e.g., China, Indonesia, and Japan) and in industrial glucoamylase production [46], [47]. As previous studies reported [48], *R. oryzae* contained a number of GH15 genes, whereas few members from families GH13 and GH31 were detected compared to the ascomycetes and basidiomycetes, which adapts to its lifestyle because storage polysaccharides do not serve as a major carbon and energy sources. *Batrachochytrium dendrobatidis*, a chytrid fungus parasitizing on amphibians, had fewer amylolytic genes and none were identified from the GH13 family.

### Branches of Amylolytic Enzymes from GH13 in the Tested Fungi Implied their Evolutionary Relationships

**Figure 1:**
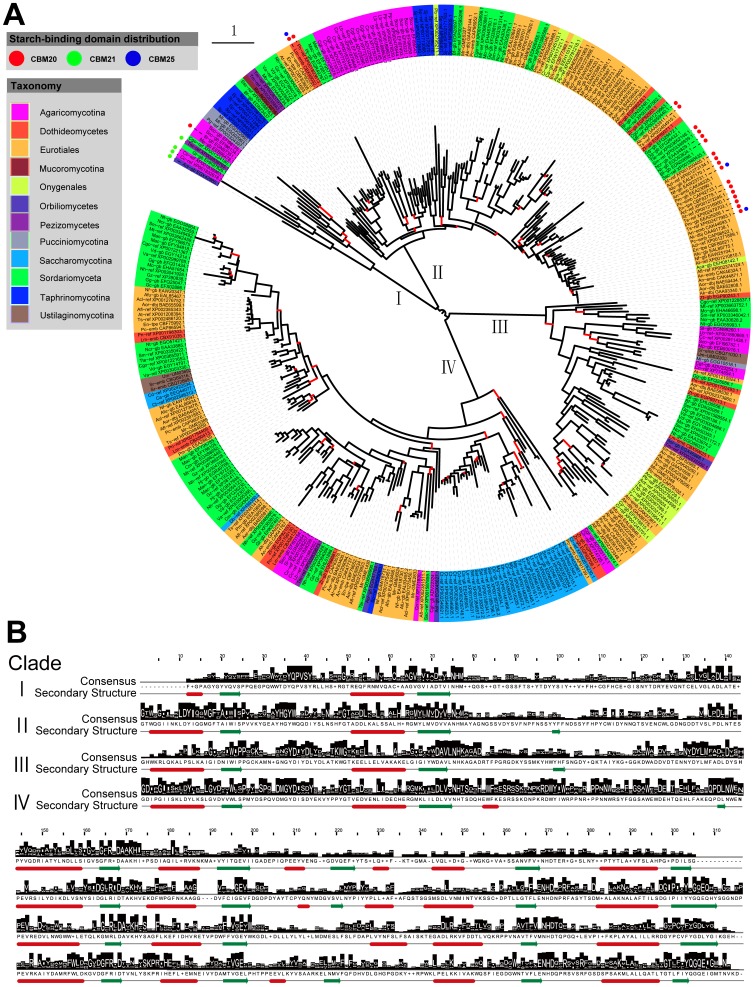
Evolutionary branches of the GH13 amylolytic enzymes from 85 fungi and their structure features. A. The inner circle was the phylogenetic tree of the GH13 amylolytic enzymes from 85 fungal genomes and the root was put at the mid-point of the longest span across the tree. The tree was inferred by FastTree from the alignments of GH13 amylolytic enzymes constructed by HMMER packages against the profile hidden Markov model of PF00128 and edited on iTOL. The bootstrap values at the inner nodes are displayed by the color that the related edges are marked in red with the values less than 800 in 1000 replicates and otherwise maintain in dark. The outer is the taxon represented as species abbreviation (shown in **Table? 1**) followed by the serial number, which is covered by different colors to show its taxonomic group as the legend indicated. Each taxon links the branch with a dotted line. Distribution of putative starch-binding domains is indicated by the scattered solid circles outside the corresponding taxon. B. Primary and secondary structure features of four clades. The consensus logos of four clades were generated by Jalview from matched residues in their alignments against the profile hidden Markov model of PF00128. In the logo, the total stack height represented the information content of amino acids at that position. The relative height of each amino acid in the stack was proportional to its frequency at the position and amino acids were sorted so the most common one was on top of the stack. Secondary structures of four consensus sequences were automatically predicted by Jpred Server embedded in Jalview that helices were marked as red tubes and sheets as dark green arrows.

The phylogeny of GH13 including α-amylases and α-glucosidases was analysed among the tested fungi and members of the GH13 family were divided into four clades for studying their protein features (**Figure 1**). In agreement with the HMM logo from α-amylase family on Pfam (http://pfam.sanger.ac.uk/family/PF00128), the primary structure analysis showed that the four clades with 316 conserved positions shared a few very well-conserved sequence regions. Among them, the residues Asp168, Glu197 and Asp271 (numbering of GH13 consensus in **Figure 1B**) forming the catalytic triad were considered totally invariantly throughout the family [49], [50]. However, four exceptions were observed. One sequence showed a deletion in the conserved Asp168 position (NCBI: XP_383879.1) and the other three sequences had Asp271 replaced with Glu, Ser and Tyr, respectively (GenBank: EGN99260.1; GenBank: CAK37367.1; NCBI: XP_001210924.1). Unfortunately, only protein CAK37367.1 was annotated as α-amylase in CAZy database (http://www.cazy.org/GH13_eukaryota.html); others were hypothetical proteins deduced from genome sequences and more *in vivo* supports are needed. In addition, a few residues, such as Tyr36, Gly49, Asp71, Asn75, His76, Arg166 and His270, were frequently present in the tested amylolytic proteins. It is worth mentioning that short sequences around His76, Asp168, Glu197 and Asp271 constituted four conserved regions of the family related to enzyme specificity, despite the overall low sequence similarity [18], [19].

Previous studies revealed that the α-amylase family shared a common catalytic domain in the form of a (β/α)_8_-barrel, a domain of eight parallel β-strands surrounded by eight α-helices [18], [51]. Secondary structure prediction of consensus sequences of four clades showed with highly conserved secondary structures in some regions and at least six of the eight helices were consistently identified (**Figure 1B**). However, these four clades also had their individual phylogenetic features, which thus may improve understanding of their phylogenetic origin.

#### Clade I: Special features in α-amylases suggest acquisition by horizontal gene transfer

Clade I with two main branches contained the fewest amount of α-amylases among the four clades. The first branch with a cluster of five putative α-amylases from the taxonomic group Agaricomycotina (2), Orbiliomycetes (1), Pezizomycetes (1) and Sordariomyceta (1) showed motif loss, containing only the first three conserved regions up to the conserved position 201. Homology searches using Blastp revealed that these putative α-amylases showed a large functional homogeneity with their animal counterparts. This was surprising, since fungal α-amylases were generally considered to be more related to each other than to the α-amylases from animals [1], [13], [14].

The putative α-amylases in the second branch were from Agaricomycotina (4), Pucciniomycotina (3) and Sordariomyceta (1). Homology searches showed that the α-amylases exhibited high sequence similarity with their counterparts from Actinomycetes. Previous studies indicated that some of the bacterial α-amylases originated from repeated horizontal gene transfer from Eukarya [13], [21]. These α-amylases with high sequence similarity from distantly related taxonomic group suggested a cause of horizontal gene transfer but the possible direction were from Actinomycetes to fungi due to the limited species range in the second branch.

#### Clade II and III: Wide presence of two distinct groups of fungal α-amylases implies their early divergence

Most of the α-amylases in the tested fungi were branched into two clades (Clade II and Clade III) based on their phylogenetic relationships. The α-amylases in each clade were from a wide range of taxonomic groups and their phylogeny was generally in agreement with their taxonomic groups such as the α-amylases in close relatives were more likely to be clustered together. Conserved domain searches of consensus sequences using Blastp against NCBI’s Conserved Domain Database showed that the catalytic domains of Clade II were recognized as similar to eukaryotic α-amylases (cd11319, E-value: 0e+00) while the catalytic domains of Clade III were recognized as similar to bacterial and fungal α-amylases (cd11318, E-value: 4.48e-163) [52]. Based on the phylogentic analysis, fungal α-amylases have been divided into two clearly distinguishable subfamilies: GH13_1 for extracellular enzymes is fungal specific while GH13_5 for intracellular enzymes is phylogentically close to the bacterial enzymes [1], [13]. It is noted that characteristics of fungal α-amylases in Clade II and III correspond to those in GH13_1 and GH13_5, respectively. Some residues recognized as GH13_5 specific are also reflected in the consensus of Clade III, including Cys27, Leu74, Tyr/Phe198, Trp199, Cys301 and Leu307 (numbering of GH13 consensus in **Figure 1B**) [13]. It is worth mentioning that more specifically conserved residues can be inferred by comparison of consensus logos from Clade II and Clade III such as Phe18, Ala20, Asn45, Met69, Tyr160, Gly186, Asp259, Asp281 and Asn288 for GH13_1 and Trp48, Ala61, AsnTyrAspTyrLeuMet130-135, Asp149, Arg247 for GH13_5 (**Figure 1**). The existence of two types of α-amylases in these fungi suggests divergent evolution of α-amylase from two sources and their divergence at a time prior to the divergence of Ascomycota and Basidiomycota since the α-amylases from both phyla were widely distributed in these two clades.

The α-amylases were also shown to occur as multiple genes in a number of the tested fungi especially in the taxonomic group Eurotiales. Close phylogenetic relationships of some α-amylases from the same species suggested an occurrence of gene duplication. Previous studies revealed gene duplications of α-amylases in many living organisms from animals, plants, fungi and bacteria [53], [54]. The evolutionary significance of the multiple genes in fungi might lie in the potential high yields of α-amylases that are relevant with the adaptation of their saprophytic lifestyle for obtaining nutrients.

#### Clade IV:GH13 α-glucosidases seem evolved from ancestral α-amylases

All annotated α-glucosidases were clustered into Clade IV. The conserved structure and catalytic mechanism within GH13 enzymes are believed to represent a common evolutionary origin [20], [55]. Phylogenetic analyses revealed that some proteins neighboring the root of Clade IV possessed an intermediate character of α-amylases and α-glucosidases, showing an ambiguous assignment due to their high sequence similarity with both enzymes. We therefore suggest that α-glucosidases evolved from ancestral α-amylases based on their gene redundancy. Generally, α-glucosidases were distributed in many species from the phyla Ascomycota and Basidiomycota but not positively identified in the selected fungi from Chytridiomycota and Zygomycota.

### Evolutionary Conservation in Glucoamylases Revealed their Importance in the Tested Fungi

**Figure 2:**
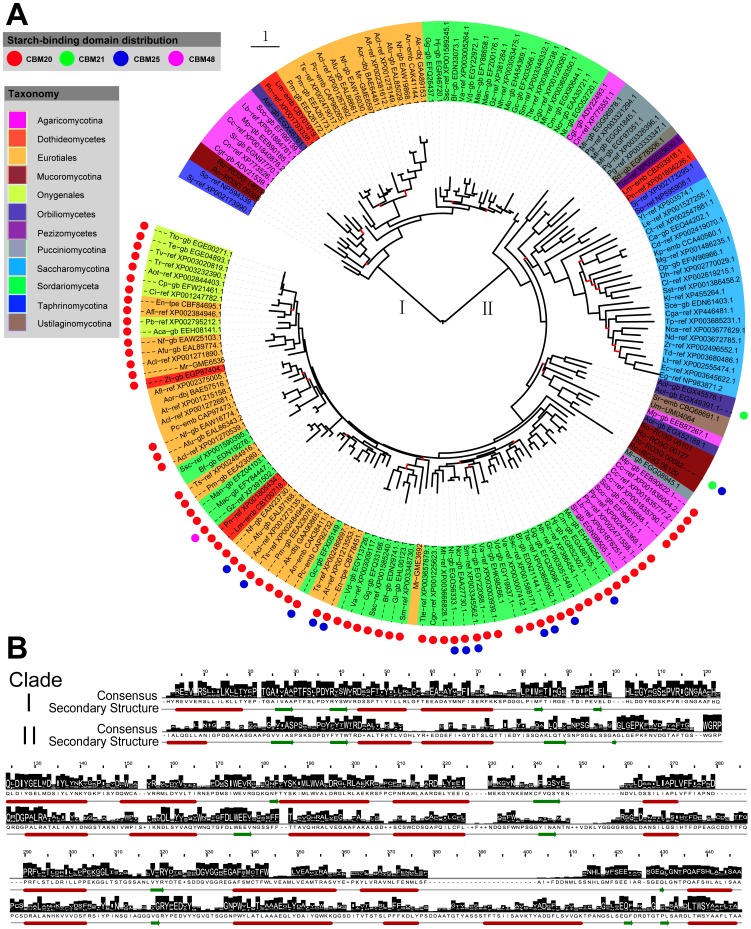
Evolutionary branches of the GH15 family from 85 fungi and their structure features. A. Phylogenetic tree of the GH15 family and B. Primary and secondary structure features of the two clades. For details see legend of Figure 1.

Members of the GH15 family from the tested fungi were divided into two clades based on their phylogenetic relationships (**Figure 2**). Primary sequence analysis revealed that the two clades shared some conserved residues. Among them, Glu175 and Glu421 (numbering of GH15 consensus in **Figure 2B**) were indentified as the two catalytic residues [23]. Most of catalytic domains from fungal glucoamylases contains 13 helices of which 12 form an (α/α)_6_-barrel [23], [25], [26]. Secondary structure prediction of consensus sequences showed that the two clades shared the conserved distribution in secondary structures. However, one helix was missing near the C-terminal segments of Clade I due to deletions in the corresponding region.

#### Clade I: Identification of a novel branch of the GH15 family

Despite the shared catalytic residues, Clade Ishowed many differences when compared to Clade II especially as some deletions in genes belonging to Clade I resulted in loss of one conserved helix as mentioned above. Moreover, homology searches using Blastp revealed that Clade I reflected an unambiguous assignment to the GH15 family without clear function. The proteins in Clade I were from a wide range of taxonomic groups involving the phyla Ascomycota, Basidiomycota and Zygomycota especially from the fungi with redundancy of glucoamylase genes. The widespread presence of these GH15 proteins suggested a specific function, currently unknown, but probably non-essential. It seems that Clade I was evolved from one of the GH15 forms existing in ancestral fungi and this form was later eliminated in many fungi with selection pressure against the other GH15 form evolved as Clade II in evolution.

#### Clade II: Glucoamylases show a conservative evolution pattern

The proteins in Clade II annotated as glucoamylases were found in all tested fungi. Generally, the phylogeny of fungal glucoamylases was divided into several main branches, probably due to the multiplicity of glucoamylase forms existing in ancestral fungi. However, fungal glucoamylases showed a conservative pattern in evolution. Glucoamylases from related species were clustered in the tree. It is worth mentioning that glucoamylases in the Saccharomycotina grouped together in the phylogenetic tree, suggesting a common evolutionary origin. This also supports the view mentioned above, namely that the fungi in the taxonomic group of Saccharomycotina were probably evolved from the common ancestral fungi. Another conserved feature of glucoamylases was reflected in their gene number. Glucoamylase genes were presented in each of the tested fungi but are maintained at relatively low number. The conserved evolution in glucoamylases reflected their important roles in fungi, and suggests that they may be essential.

### Multiple Branches of GH31 α-glucosidases Suggested their Diverse Evolutionary Paths

**Figure 3:**
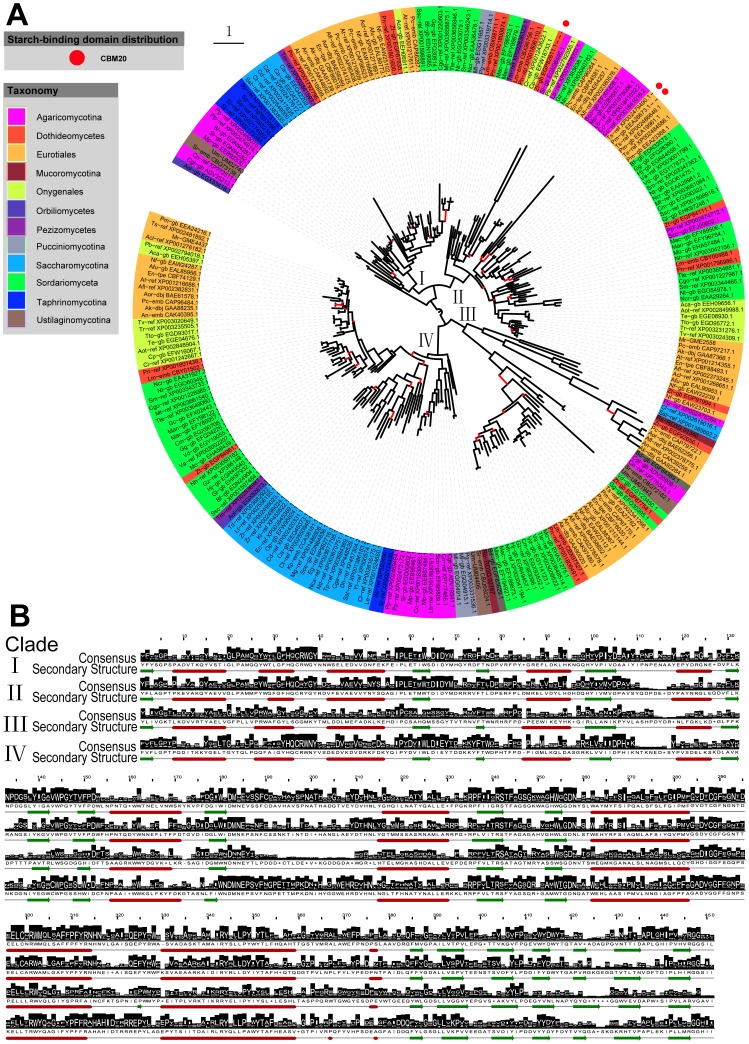
Evolutionary branches of the GH31 α-glucosidases from 85 fungi and their structure features. A. Phylogenetic tree of GH31 α-glucosidases and B. Primary and secondary structure features of the four clades. For details see legend of Figure 1.

These enzymes were divided into four major clades on the basis of sequence comparisons (**Figure 3**). Interestingly, there was a putative α-glucosidase (GenBank: EGX53418.1) outside the four clades that appeared to be rather unique. Homology searches using Blastp revealed that the conservative domain of this protein was distantly related to their animal and plant counterparts.

Primary structural analyses of GH31 α-glucosidases in the tested fungi displayed some characteristic residues. Among them, the invariant Asp182 (nucleophile) and Asp257 (acid/base) (numbering of GH31 consensus in **Figure 3B**) have been identified as the catalytic residues [30], [33], [56]. Previous studies revealed a characteristic sequence motif of GH31 α-glucosidases with the signature DMNE (position 182–185 in the logo) in the region surrounding the catalytic nucleophile [30]. However, another sequence motif of GH31 α-glucosidases was found in the same region that Clade III showed as the signature DNNE. Variations in this region seemed to reflect the early divergence of Clade III from the other GH31 α-glycosidases in the evolutionary process [30]. Comparative analyses of secondary structures indicated that a common scaffold was conserved throughout the family. However, a number of subgroups in GH31 α-glucosidases in view of their phylogenetic relationships suggested that GH31 α-glucosidases had undergone diverse evolutionary paths.

#### Clade I and II: Two branches of lysosomal acid α-glucosidases

Conserved domain searches of both consensus sequences revealed specific matches to lysosomal acid α-glucosidases (cd06602, E-value: 0e+00). It is worth mentioning that the enzymes in these two clades were all from a wide range of taxonomic groups. This widespread presence suggests multiple forms of lysosomal acid α-glucosidases in ancestral fungi.

#### Clade III: Phylogenetically related to bacterial α-glucosidases

As mentioned above, Clade III (with two main branches) suggested a different evolutionary process in view of the new signature surrounding the catalytic nucleophile. In the upper branch, the putative α-glucosidases reflected a close phylogenetic relationship with their bacterial counterparts based on homology searches, some of which, such as from the taxonomic group Eurotiales, were with specific hits to the bacterial α-glucosidases (cd06594). As these enzymes are present in a few species, they may have been horizontally transferred from bacteria.

The putative α-glucosidases in the other branch of Clade III came from a wide range of fungi including the Ascomycota, Basidiomycota and Chytridiomycota. Homology searches revealed that these enzymes were phylogenetically related to their bacterial counterparts. But their catalytic domains showed non-specific hits to current identified groups in NCBI’s Conserved Domain Database. Probably, these enzymes belonged to a new clade with the signature of DNNE adjacent to the catalytic nucleophile.

#### Clade IV: A large branch evolved as neutral α-glucosidases

The conserved domain of Clade IV showed matches to neutral α-glucosidases (cd06603, E-value: 0e+00). The putative α-glucosidases belonging to this large branch were positively identified in all the tested taxonomic groups. Moreover, the phylogeny of α-glucosidases in this branch was highly in agreement with their taxonomic relationships. This suggests that this α-glucosidase clade is evolutionarily conserved and may be essential in fungi.

### Distribution of Starch-binding Domains Seems Related to Fungal Taxonomy and Amylase Phylogeny

About 10% of microbial amylolytic enzymes contain starch-binding domains appended to catalytic modules to mediate the binding of raw starch [40], [42]. For better understanding of the amylase architectures, we surveyed the distribution of CBM20, CBM21, CBM25 and CBM48 in the annotated enzymes. The putative domains were identified from the annotated enzymes by HMMER searches.

**Figure 4:**
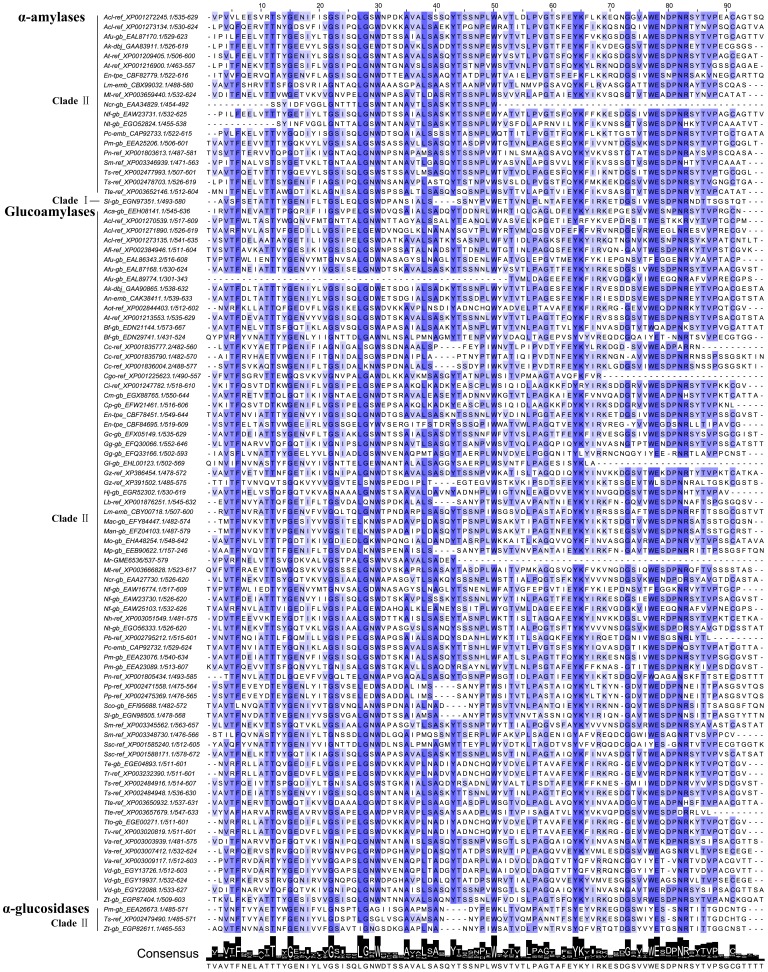
Sequence alignments of putative proteins from CBM family 20. Multiple alignments of putative proteins were performed by aligning them to the profile hidden Markov model of PF00686 with HMMER package. Residues assigned to match states were reserved for the profile analysis and their consensus logo and numbering were generated by Jalview. Protein sequence ID is represented as species abbreviation followed by serial number and domain position.

The family CBM20 is known as a classical C-terminal starch-binding domain of microbial amylases [57]. Our investigation showed that CBM20 occurs in some GH13 α-amylases (about 9%) and most GH15 glucoamylases (about 51%). However, several CBM20s were found in GH31 α-glucosidases (**Figure 4**). The binding ability of CBM20s to starch seems to be associated with certain consensus residues despite no invariant residues in the family [37]. There are two separate glucan-binding sites in CBM20s. Binding site 1 consists of Trp30, Lys65, Trp77, Glu78 and Asn82, and binding site 2 is defined by Thr12, Tyr14, Gly15, Glu16, Asn17, Asp41, Tyr43 and Trp50 (numbering of CBM20 consensus in **Figure 4**) [37]. However, it is noted that some residues in binding positions such as Tyr14, Glu16, Asn17 and Asp41 are not well-conserved. Besides, alignment analysis revealed additional residues Phe6, Gly22, Leu27, Gly28, Ala35, Leu38, Ala40, Tyr64, Gly73 and Arg83 with high percentage identity in fungal amylolytic enzymes.

**Figure 5:**
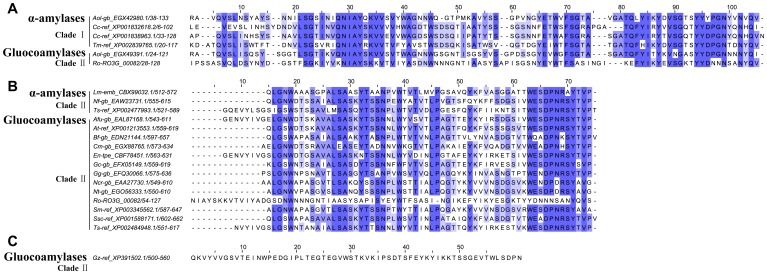
Sequence alignments of putative proteins from CBM families 21, 25 and 48. A, B and C correspond to the alignments of CBM21, 25 and 48 adjusted against the profile hidden Markov models of PF03370, PF03423 and PF02922 respectively.

The family CBM21 is known as the N-terminally positioned starch-binding domain of *Rhizopus* glucoamylase [58]. A few CBM21s were found in GH13 α-amylases and GH15 glucoamylases (**Figure 5A**). Two cooperative raw starch-binding sites have been elucidated in *R. oryzae* glucoamylase. Binding site 1 (responsible mainly for binding) involves the residues Trp45, Tyr84 and Tyr94, whereas binding site 2 (responsible mainly for facilitating binding) contains the key residues Tyr32 and Tyr65 (numbering of CBM21 consensus in **Figure 5A**) [57], [59].

The CBM25 family was established based on revealing a novel type of starch-binding domain with two copies in a bacterial α-amylase [57], [60]. The putative domains were hit upon some GH13 α-amylases and GH15 glucoamylases (**Figure 5B**). However, it seems that all putative domains presenting in a single copy are within the region of corresponding CBM20s, except one from *R. oryzae* glucoamylase shows its domain within CBM21. It’s unclear whether these CBM20s and CBM21 have the CBM25 motif. Anyhow, it reflected a close phylogenetic relationship between them.

The CBM48 family was established containing the putative starch-binding domains from the pullulanase subfamily [61]. Only one putative domain was detected in a GH15 glucoamylase (**Figure 5C**). However, this domain also overlaps with the CBM20. Further analysis showed that distribution of starch-binding domains seems related to fungal taxonomy and amylase phylogeny.

#### Amylolytic enzymes with starch-binding domains are concentrated in filamentous fungi from Ascomycota

In our analysis, amylolytic enzymes with starch-binding domains were merely from filamentous fungi. No hits of four domains were showed in amylolytic enzymes from the tested yeasts and mushrooms. Interestingly, except the glucoamylase from *R. oryzae*, amylolytic enzymes with starch-binding domains were concentrated in filamentous fungi belonging to the phylum Ascomycota. The limited spread of starch-binding domains may also support their isolated phylogeny [36], [42].

#### Amylolytic enzymes containing starch-binding domains are phylogenetically related

Starch-binding domains have been revealed an independent evolution to the catalytic domains [36], [42]. However, it is noted that amylolytic enzymes with starch-binding domains in each family show close evolutionary relationships based on their catalytic domains. In GH13 family, the enzymes containing starch-binding domains were clustered in Clade I (heterologous α-amylases) and Clade II (extracellular fungal α-amylases) (**Figure 1**). Obviously, glucoamylases with starch-binding domains were clustered in one branch of Clade II (**Figure 2**). In GH31 family, the enzymes with starch-binding domains were gathered in Clade II (**Figure 3**). All suggest relevance of amylase phylogeny and starch-binding domain distribution. It implies that acquisition of starch-binding domains may occur in certain phylogenetic groups [36].

### Conclusions

In this study, the genomic distribution, architecture and phylogeny of amylolytic enzymes including α-amylase, gluocoamylase and α-glucosidase in the available genomes of 85 fungal strains were investigated. Genomic distribution of amylolytic genes suggests their adaptation to the lifestyles of the fungi, at least with respect to starch degradation. Evolutionary significance of the adaptation may lie in their mode of survival, especially in saprobism for obtaining nutrients. Putative starch-binding domains of CBM20, CBM21, CBM25 and CBM48 are concentrated in phylogenetically related amylolytic enzymes from filamentous fungi, especially in Ascomycota. It supports the separate evolution of starch-binding domains to the individual enzymes and suggests their acquisition occurring in certain phylogenetic groups of amylolytic enzymes.

**Figure 6:**
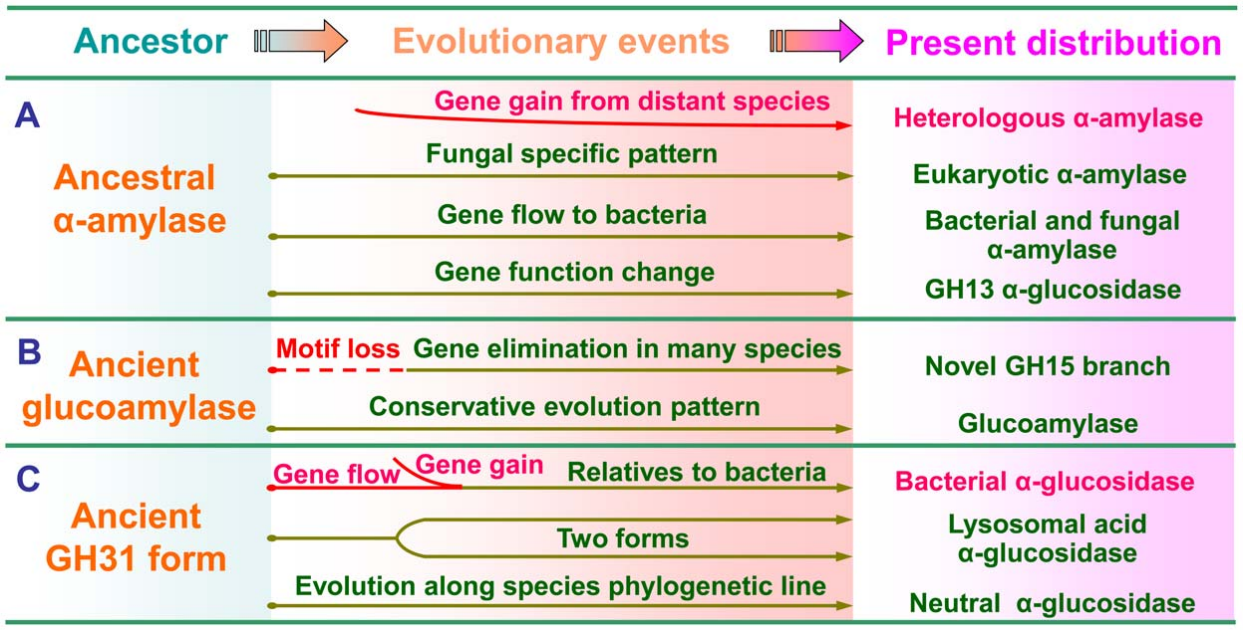
Possible evolutionary scenarios for amylolytic enzyme evolution in fungi. A. Evolutionary scenarios for the GH13 enzymes. A few α-amylases identified as heterologous α-amylases might be transferred from animals and Actinomycetes. Eukaryotic, bacterial and fungal α-amylases correspond to subfamilies GH13_1 and GH13_5, respectively. GH13 α-glucosidases seem evolved from ancestral α-amylase. B. Evolutionary scenarios for the GH15 enzymes. The function of novel GH15 branch is currently unknown. C. Evolutionary scenarios for the GH31 enzymes. The enzymes in the group of temporarily named bacterial α-glucosidase are phylogenetically close to their bacterial counterparts. They may constitute a new clade of GH31 α-glucosidases in fungi.

Phylogenetic analyses showed evidence for likely evolutionary events, such as horizontal gene transfer, gene duplication, and gene loss for amylolytic enzymes. We raised a hypothetical scheme for the evolution of genes encoding amylolytic enzymes in fungi (**Figure 6** **)**. GH13 amylolytic enzymes that originated from a common ancestor were evolved into three branches prior to the divergence of Ascomycota and Basidiomycota. Among the two branches of α-amylases, one maintaining the fungal style was developed as the clade of eukaryotic α-amylases, the other evolving as the bacterial and fungal α-amylases was transfered to bacteria as an important origin of bacterial α-amylases. It is worth mentioning that the α-amylase genes might be lost in the ancestor of the Saccharomycotina, resulting in their relatively poor capability for starch hydrolysis. Gluocoamylase genes were identified in all tested fungi and showed conserved evolution, probably because they are essential in fungi. The novel GH15 branch in some species might be derived from the motif loss of an ancient gluocoamylase version. This version was later eliminated in many fungi with selection pressure since it may have been dispensable for function in fungi. GH31 α-glucosidases seemed to experience diverse evolutionary paths. Among them, the clade of neutral α-glucosidases showed conservation along phylogenetic lines. Lysosomal acid α-glucosidases, constituting another large extant clade are suggested to be evolved from two forms of lysosomal acid α-glucosidases existing in ancestral fungi. Bacterial α-glucosidases were identified as a new clade of GH31 α-glucosidases in fungi, which seemed to have arisen from two origins in response to their phylogenetic relationships with their bacterial counterparts. One was attributed to gene flow to bacteria, and the other seemed to have resulted from horizontal gene transfer from bacteria to fungi. Our results provide new insights that will be valuable for the understanding of evolutionary relationships in the major subgroup of amylolytic enzymes in fungi. Meanwhile, it also provides some clues on investigating fungal evolutionary adaptation to the ecological conditions in the view of their diversification in starch degrading ability.

## Materials and Methods

### Sequence Data

Overall protein sequences of 85 strains of fungi from the phyla Ascomycota, Basidiomycota, Chytridiomycota and Zygomycota were used in this study (Table 1).

### Annotation of Amylolytic Genes

The annotation pipeline of amylolytic genes in selected fungi was in a two-step procedure of identification and annotation. The identification step of the families GH13, GH15 and GH31 was performed by using HMMER 3.0 (http://hmmer.janelia.org/) with hmmsearch of profile hidden Markov models derived from the Pfam seed alignment flatfiles of PF00128 (GH13), PF00723 (GH15 ) and PF01055 (GH31) (downloaded from the Pfam protein families database, http://pfam.sanger.ac.uk/) against fungal overall protein sequences. The hits passed MSV, Bias, Vit and Fwd filters (see HMMER User’s Guide, http://eddylab.org/) were then subject to the annotation procedure involving BlastP comparisons against the database of non-redundant protein sequences (http://blast.ncbi.nlm.nih.gov/Blast.cgi). Based on high levels of similarity and/or a large functional homogeneity of the hits, these predicted amylolytic enzymes were annotated as α-amylases, glucoamylases and α-glucosidases.

### Survey of Starch-binding Domains in the Annotated Amylolytic Enzymes

Distribution of four carbohydrate-binding module families CBM20, CBM21, CBM25 and CBM48 involving in starch binding was surveyed in the annotated amylolytic enzymes. Profile hidden Markov models of PF00686 (CBM20 family), PF03370 (CBM21 family), PF03423 (CBM25 family) and PF02922 (CBM48 family) from Pfam database were used for HMMER searching against all annotated enzymes. The hits passed MSV, Bias, Vit and Fwd filters were selected as the putative domains.

### Construction of Phylogentic Trees

Alignment of amino acid sequences in the GH13, GH15 and GH31 families were carried out by HMMER package against the corresponding profile hidden Markov models. Phylogenetic trees from alignments of protein sequences were constructed by FastTree version 2.1.4 by maximum likelihood methods (http://www.microbesonline.org/fasttree/) [62]. The tree data were submitted to iTOL (http://itol.embl.de/upload.cgi) for viewing phylogenetic trees and making figures [63].

### Structural Feature Analysis of Protein Sequences

In this study, structural features were explored in groups of homologous proteins based on their phylogenetic relationships to reveal subfamily-specific conservation patterns, essentially conserved within each subfamily but differing across subfamily. Multiple protein sequence alignments built by HMMER package were edited by Jalview version 2.7 [64]. And residues assigned to match states that conserved against the Pfam annotations were reserved for the profile analysis.

Consensus logos automatically generated by Jalview were used for visualization of the conservation of primary structure by plotting a stack of amino acids for each position. Secondary structures of consensus sequences extracted from the alignments were predicted by Jpred Server version 3.0.1 embedded in Jalview to exploit evolutionary information from multiple sequences [65].
